# Determinants of Change in Glycemic Status in Individuals with Prediabetes: Results from a Nationwide Cohort Study in Germany

**DOI:** 10.1155/2018/5703652

**Published:** 2018-10-14

**Authors:** Rebecca Paprott, Christa Scheidt-Nave, Christin Heidemann

**Affiliations:** Department of Epidemiology and Health Monitoring, Robert Koch Institute, 12101 Berlin, Germany

## Abstract

Previous studies investigating determinants of changes in glycemic status among individuals with prediabetes mainly focused on glucose-defined prediabetes. In this study, we examined determinants of a regression to normoglycemia or a progression to diabetes among individuals with HbA1c-defined prediabetes. The study included 817 participants (18–79 years) with prediabetes (HbA1c 5.7–6.4% (39–47 mmol/mol)) at baseline. Glycemic status at follow-up was categorized as diagnosed diabetes (self-reported physician diagnosis or antidiabetic medication), undiagnosed diabetes (HbA1c ≥ 6.5% (≥48 mmol/mol)), prediabetes (as defined at baseline), and normoglycemia (HbA1c < 5.7% (<39 mmol/mol)). Determinants of glycemic changes were identified by multinomial logistic regression (OR (95% CI)), with those remaining in the prediabetic state as reference. During a mean follow-up time of 12.0 years, 33.8% of the participants reverted to normoglycemia, 7.2% progressed to undiagnosed diabetes, 12.8% progressed to diagnosed diabetes, and 46.2% remained prediabetic. Determinants of a regression to normoglycemia were female sex (male vs. female: 0.67 (0.46; 0.98)) and higher HDL cholesterol levels (1.17 (1.02; 1.35) per 10 mg/dl). Determinants of a progression to undiagnosed or diagnosed diabetes were higher values of BMI (1.10 (1.02; 1.18); 1.13 (1.06; 1.21) per kg/m^2^), waist circumference (1.04 (1.01; 1.07); 1.06 (1.03; 1.09) per cm), alanine aminotransferase (1.06 (1.03; 1.09); 1.07 (1.03; 1.10) per U/l), and gamma-glutamyl transferase (1.02 (1.00; 1.03); 1.03 (1.01; 1.04) per U/l). Higher age (1.04 (1.02; 1.06) per year), female sex (male vs. female: 0.56 (0.33; 0.97)), and parental history of diabetes (yes vs. no: 1.82 (1.05; 3.15)) were further associated with a progression to diagnosed diabetes, whereas higher triglyceride levels (1.03 (1.01; 1.06) per 10 mg/dl) were associated with a progression to undiagnosed diabetes. In conclusion, among the investigated determinants, potentially modifiable anthropometric and metabolic markers were associated with glycemic changes in individuals with HbA1c-defined prediabetes. The findings of this study demonstrate the need for more refined case finding strategies for diabetes prevention.

## 1. Introduction

Type 2 diabetes is a chronic metabolic disease characterized by elevated blood glucose levels that increase the risk for severe micro- and macrovascular complications [1]. Prediabetes is considered a prestage of manifest diabetes and is associated with an increased risk of developing type 2 diabetes [2–5]. Approximately 5% to 10% of individuals with prediabetes progress to diabetes yearly [2]. However, progression can be prevented by lifestyle and pharmacological interventions and prediabetes may even revert to normoglycemia [2, 3].

Prediabetes can be defined by impaired fasting glucose (IFG) (5.6–6.9 mmol/l), impaired glucose tolerance (IGT) (7.8–11.0 mmol/l), or elevated HbA1c levels (5.7–6.4% (39–47 mmol/mol)) [5]. Previous studies on determinants of progression or regression from prediabetes were mostly based on prediabetes defined by IFG or IGT, whereas studies using elevated HbA1c levels for definition are sparse.

In these studies, among individuals with IFG or IGT, measures of lipid metabolism [6–10] and anthropometry [4, 8, 11–14] were associated with changes in glycemic status. Moreover, several previous studies among persons with IFG or IGT examined the effect of modifiable lifestyle factors (mostly physical activity, smoking, and alcohol intake) on glycemic changes but the majority did not find an association [4, 6–10, 15–17]. In addition, a recent study that included individuals with HbA1c-defined prediabetes found no association between physical activity and a regression to normoglycemia [17].

Since the characteristics of individuals with prediabetes who are diagnosed by different criteria may vary due to differences in the underlying pathophysiological pathways [2, 18], determinants of changes in glycemic status may also differ according to the applied diagnostic criterion.

In the present study, we therefore aimed to identify determinants of a regression to normoglycemia or a progression to undiagnosed or diagnosed diabetes in a nationwide sample of German adults with HbA1c-defined prediabetes who were followed for 12 years. In addition to sociodemographic characteristics, we investigated potentially modifiable risk factors such as anthropometric, metabolic, and lifestyle factors as determinants of change in glycemic status.

## 2. Materials and Methods

### 2.1. National Health Examination Surveys

**Figure 1:**
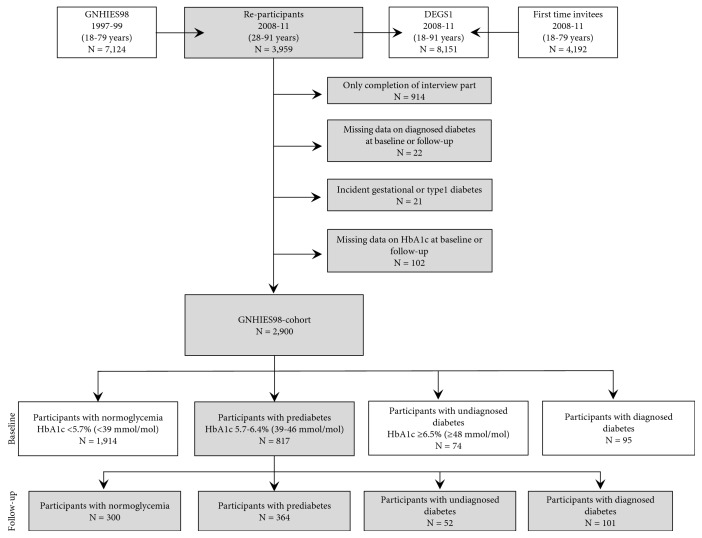
Flow chart showing the definition and distribution of participants within categories of glycemic status. Definitions applied for glycemic status were the same at baseline and follow-up. The numbers shown were drawn from the unimputed data set.

The “German National Health Interview and Examination Survey 1998” (GNHIES98) was conducted from 1997 to 1999 and targeted the residential German population aged 18 to 79 years (response: 61%). A two-stage cluster sampling procedure was applied. First, sample points were selected, and then, age- and sex-stratified random samples from local population registries were drawn [19]. Out of all GNHIES98 participants, 3959 participants (response: 62%) also took part in the subsequent “German Health Interview and Examination Survey for Adults” (DEGS1), conducted from 2008 to 2011 (Figure 1) [20]. Both surveys were conducted in accordance with the Declaration of Helsinki and approved by the Federal and State Commissioners for Data Protection. DEGS1 was approved by the ethics committee of the Charité-Universitätsmedizin Berlin (No. EA2/047/08). Participants provided written informed consent before participation [20].

### 2.2. Study Population

Figure 1 depicts a flow chart reflecting the composition of the study population. After applying different exclusion criteria among those who participated in both GNHIES98 (baseline) and DEGS1 (follow-up), a cohort consisting of 2900 participants remained. The final study sample comprised 817 participants with prediabetes at baseline.

### 2.3. Assessment of Glycemic Status

Diagnosed diabetes was determined by a self-reported history of diagnosed diabetes assessed in physician-administered standardized interviews or by the intake of antidiabetic agents within the 7 days prior to the interview documented through a detailed medication review. Among participants without diagnosed diabetes, normoglycemia (HbA1c < 5.7% (<39 mmol/mol)), prediabetes (HbA1c 5.7–6.4% (39–47 mmol/mol)), and undiagnosed diabetes (HbA1c ≥ 6.5% (≥48 mmol/mol)) were defined according to recent ADA recommendations [5]. In GNHIES98, HbA1c was measured using a Diamat high-performance liquid chromatography (HPLC) analyzer (Bio-Rad Laboratories, Munich, Germany) and RECIPE reagents (RECIPE Chemicals and Instruments, Munich, Germany). In DEGS1, HbA1c was measured using an immunoturbidimetric method (ARCHITECT ci8200; Abbott, Wiesbaden, Germany). Both methods were traceable to the National Glycohemoglobin Standardization Program [21], and no systematic deviation of HbA1c measurements between the surveys was evident [22].

### 2.4. Assessment of Risk Factors for Type 2 Diabetes

The assessment of several health-related risk factors in GNHIES98 has been previously described in detail [20, 22–25]. Briefly, information about educational level, sport activity, smoking status, residential traffic intensity, and mental distress was obtained through a standardized self-administered questionnaire. For the current analyses, middle and high educational levels were combined. Sport activity was ascertained as regular sport activity over the past three months and categorized as either no sport or any sport. Smoking status was categorized into never, former, and current smoker. Residential traffic intensity was dichotomized into rare to considerable traffic versus heavy to extreme traffic. Psychosocial distress was measured by the Mental Health Inventory (MHI-5) on a scale ranging from 0 to 100 points, with lower values indicating mental distress and higher values indicating mental well-being. A parental history of diabetes was assessed in DEGS1 through a standardized physician-administered personal interview. In GNHIES98, measures of body height, weight, and waist circumference were obtained while participants wore light clothing. BMI was calculated as the ratio of body weight (kg) and height squared (m^2^).

The intake of coffee, red meat, and whole grains (including whole grain bread, buns, and muesli) (as components of the German Diabetes Risk Score (GDRS) that is described below) was assessed through a questionnaire on frequency of consumption of these food groups. The average amount consumed per day was estimated by further considering information on dietary intake assessed through a diet history method in a GNHIES98 subsample (*n* = 4030) as previously described [23].

As a summary measure of overall risk for type 2 diabetes, we calculated the GDRS including age, height, waist circumference, history of hypertension, physical activity, smoking, family history of diabetes, and intake of coffee, red meat, and whole grains as score components [26]. The GDRS has been shown to be a valid tool for prediction of diagnosed diabetes in the general adult population [23].

Venous blood samples were taken, processed within an hour, and stored at −40°C in the central laboratory unit at the Robert Koch Institute until further analyses. HDL cholesterol was determined based on the cholesterol oxidase-peroxidase 4-aminophenazone method (MEGA, Merck, Darmstadt, Germany) (intra-assay CV: 1.2–2.5%). Triglycerides were determined by the glycerol-3-phosphate oxidase-peroxidase 4-aminophenazone method using the MEGA measurement device (Merck, Darmstadt, Germany) (intra-assay CV: 1.6–2.2%). Both gamma-glutamyl transferase (GGT) and alanine aminotransferase (ALT) were measured according to standards of the DGKC (German Association of Clinical Chemistry) (EPOS, Eppendorf, Wesseling, Germany) (intra-assay CV for GGT: 1.3–2.7%; for ALT: 1.1–4.8%). High-sensitivity C-reactive protein (hs-CRP) was measured in serum by an immunoturbidimetric method (ARCHITECT ci8200, Abbott, Munich, Germany) (intra-assay CV: 4.0).

Additionally, diagnosed hypertension, hyperlipidemia, myocardial infarction, and stroke were considered as comorbidities and assessed through a physician-administered personal interview. The use of statins and thiazide diuretics was considered as concomitant therapy known to favor the progression from prediabetes to diabetes and was ascertained through a detailed medication review. However, we found that the number of participants taking thiazide diuretics (*n* = 20) or statins (*n* = 32) at the time of baseline examination (1997–99) was too low to enable further analyses.

### 2.5. Statistical Analysis

The SAS software 9.4 (SAS Institute Inc., Cary, NC) was used for all statistical analyses. A weighting factor was applied that corrects for deviations of the DEGS1 sample from population figures from the Federal Statistical Office (as of 31 December 2010) and accounts for the incomplete follow-up [27]. For the current analyses, the probability of GNHIES98 participants with prediabetes participating in DEGS1 was derived from a generalized linear mixed model with age at the time of GNHIES98 (4 categories) and age at the time of DEGS1 (8 categories), as well as smoking (yes or no), education (3 categories), income (3 categories), and migration background (yes or no) at the time of GNHIES98, as independent variables.

Multinomial logistic regression was performed to calculate the odds ratios (OR) and 95% confidence intervals (CI) for considered risk factors. For the dependent variable, three categories were defined according to a participant's glycemic status at follow-up, with those who remained in the prediabetic state as the reference group. In the case of a regression to normoglycemia, an OR > 1.00 indicates a higher chance for a regression (i.e., favorable). In the case of a progression to diabetes, an OR > 1.00 indicates a higher chance of a progression (i.e., unfavorable).

We classified a total of 19 potential determinants (Table 1) into the following categories and subcategories: (1) *unmodifiable diabetes risk factors* comprising sociodemographic factors and family history of diabetes and (2) *potentially modifiable risk factors*, including anthropometric markers, lifestyle factors, residential traffic intensity, mental distress, the diabetes risk score (as a summary measure of predominantly modifiable factors), and metabolic markers. Risk factors were examined in separate multinomial regression models, adjusting for age and sex (model 1). For potentially modifiable risk factors, a second model (model 2) was fitted that additionally adjusted for educational level and other potential confounders that were selected by applying directed acyclic graphs [28].

Among individuals with prediabetes at baseline, 22% had missing data in at least one of the considered determinants. To account for the missing values, multiple imputation was applied that included all potential determinants, glycemic status at follow-up, the weighting factor, and the cluster variable. The SAS procedure “PROC MI” with the fully conditional specification method for an arbitrary pattern of missingness was used, and 25 complete data sets were imputed [29, 30]. The fraction of missing information ranged from <0.001 for height to 0.08 for hs-CRP. The relative efficiency was >0.99 for all risk factors imputed. SAS survey procedures were also applied to the individual imputed data sets again using the weighting factor and the cluster variable [29]. Results from the individual imputed data sets were then combined using the SAS procedure “PROC MIANALYZE,” which accounts for the variability between the results of imputed data sets [31].

A considerable proportion of participants had an HbA1c level of 5.7% (39 mmol/mol; equivalent to 26.1%) or of 6.4% (47 mmol/mol; equivalent to 2.4%), i.e., an HbA1c level that was borderline for the definition of normoglycemia or diabetes, respectively. Therefore, *relevant* changes in glycemic status were defined for a sensitivity analysis as a change in the category of glycemic status combined with a change of at least ±0.3 percentage points in HbA1c. This definition of a *relevant* glycemic change was adapted from Kowall et al., who defined relevant changes in glycemic status based on fasting or 2 h glucose [12].

In additional analyses, the associations between changes in modifiable risk factors during follow-up and changes in glycemic status were examined. A change in a modifiable risk factor was defined as the measurement at follow-up minus the measurement at baseline. In addition to models 1 and 2 described above, a third model (model 3) was analyzed additionally including the baseline value of the respective risk factor.

## 3. Results

During a mean follow-up time of 12.0 years, 7.2% of the 817 participants with prediabetes at baseline progressed to undiagnosed diabetes and 12.8% progressed to diagnosed diabetes. During the same period, 33.8% reverted to normoglycemia and 46.2% remained prediabetic (Table 1). When considering *relevant* changes in glycemic status, the proportion of participants who reverted to normoglycemia was 28.1% (95% CI: 22.5–34.4%), while 51.9% (46.2–57.6%) remained in the prediabetic stage. The proportion of participants with a progression to undiagnosed or diagnosed diabetes remained unchanged (data not shown).

Baseline characteristics of participants according to changes in glycemic status during follow-up are shown in Table 1. Compared to those who remained in the prediabetic state, participants who regressed to normoglycemia had lower values for BMI, waist circumference, triglycerides, GGT, and the diabetes risk score. Additionally, they showed higher levels of HDL cholesterol. On the other hand, participants who progressed to diagnosed or undiagnosed diabetes were older and had higher values for BMI, waist circumference, triglycerides, GGT, ALT, and the diabetes risk score. Those who progressed to diagnosed diabetes were also more often female, living in streets with heavy or extreme residential traffic intensity, of a low educational level, and characterized by a lower intake of red meat and a higher level of hs-CRP. Those who progressed to undiagnosed diabetes were less likely to be current smokers and had a lower intake of whole grains compared to those who remained prediabetic.

Additional descriptive analyses considering diagnosed comorbidities at baseline (hypertension, hyperlipidemia, myocardial infarction, and stroke) showed that, as expected, participants with a progression to diagnosed diabetes were more likely and those with a regression to normoglycemia were less likely to have been diagnosed with hypertension or hyperlipidemia than those who remained prediabetic. No significant differences across categories of glycemic changes were observed with respect to myocardial infarction and stroke (data not shown).

Table 2 depicts the association between the investigated baseline determinants and changes in glycemic status as determined from multivariate analyses. Among the unmodifiable risk factors, female sex was associated with a regression to normoglycemia and a progression to diagnosed diabetes. Higher age and a parental history of diabetes were associated with a progression to diagnosed diabetes. Among the potentially modifiable risk factors, higher levels of HDL cholesterol and lower values of the diabetes risk score were associated with a regression to normoglycemia. Higher values of BMI, waist circumference, GGT, ALT, and the diabetes risk score were associated with a progression to both undiagnosed diabetes and diagnosed diabetes. In addition, a higher triglyceride level was associated with a progression to undiagnosed diabetes. The lifestyle factors considered, residential traffic intensity, and mental distress were not individually associated with a change in glycemic status. The results persisted when analyses were repeated considering only *relevant* glycemic changes (data not shown).

Further analyses of changes in potentially modifiable risk factors between baseline and follow-up (Supplementary Table 1) revealed that decreases in the levels of BMI (OR: 0.86 (95% CI: 0.77–0.95)), waist circumference (0.96 (0.93–0.99)), and triglycerides (0.94 (0.89–1.00)) during follow-up were associated with a regression to normoglycemia. However, an increased waist circumference (1.04 (1.00–1.08)) was associated with a progression to undiagnosed diabetes whereas an increased triglyceride level (1.05 (1.02–1.09)) was related to a progression to diagnosed diabetes. An increase in the diabetes risk score during follow-up was associated with a progression to undiagnosed diabetes (1.04 (1.01–1.08)) and to diagnosed diabetes (1.06 (1.01–1.10)).

## 4. Discussion

**Figure 2:**
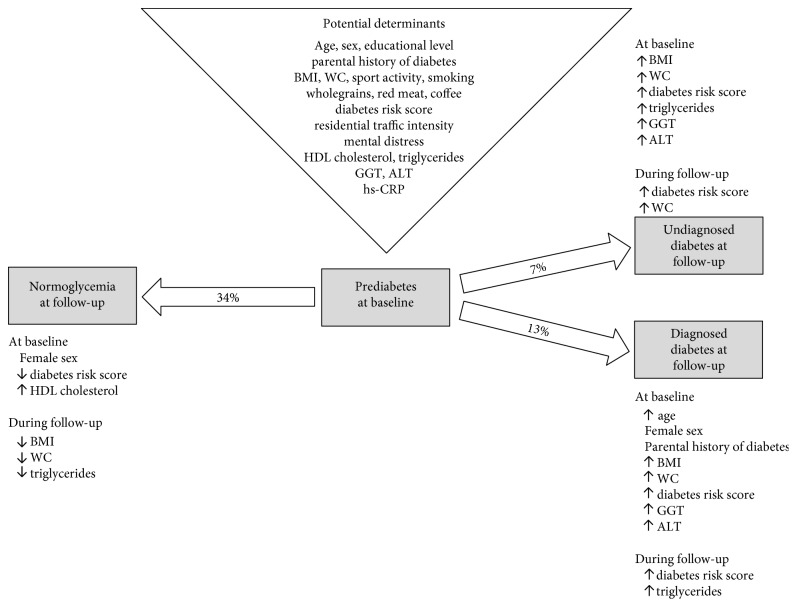
Overview on hypothesized determinants and study results. ↑ is equivalent to “higher at baseline/increase during follow-up”; ↓ is equivalent to “lower at baseline/decrease during follow-up”; BMI: body mass index; WC: waist circumference; HDL: high-density lipoprotein; GGT: gamma-glutamyl transferase; ALT: alanine aminotransferase; hs-CRP: high-sensitivity C-reactive protein.

After 12.0 years of follow-up, 20% of HbA1c-defined prediabetic adults from a nationwide German study had progressed to diagnosed or undiagnosed diabetes, whereas 46% had remained prediabetic and 34% had reverted to normoglycemia.  Figure 2 provides an overview of the hypothesized determinants of the study and its findings. Among the unmodifiable risk factors, female sex was related to a higher chance of a regression to normoglycemia and a progression to diagnosed diabetes. Additionally, older age and a parental diabetes history were related to a higher chance of a progression to diagnosed diabetes. Among potentially modifiable risk factors considered in the current study at baseline, favorable HDL cholesterol levels were related to a regression to normoglycemia. Less favorable levels of anthropometric markers and liver enzymes were associated with a progression to undiagnosed diabetes and diagnosed diabetes. In addition, less favorable triglyceride levels were related to a progression to undiagnosed diabetes. Higher values of the diabetes risk score as a summary measure of predominantly modifiable risk factors were associated with a progression to undiagnosed diabetes and diagnosed diabetes, whereas lower values were related to a regression to normoglycemia.

### 4.1. Discussion of Main Results

In this cohort, women had a higher chance of both a regression to normoglycemia and a progression to diagnosed diabetes. This finding might reflect a higher utilization of healthcare and greater health consciousness among women compared to men [32]. Higher age was associated with progression to diagnosed diabetes, confirming the well-known positive association between aging and the risk of type 2 diabetes. In addition, our findings related to a parental history of diabetes are consistent with findings from previous studies, indicating that individuals with a family history of diabetes are more likely to have their glucose level tested [33] and that a family history of diabetes probably affects an individual's knowledge of having diabetes [34].

Our findings regarding BMI and waist circumference mostly comport with findings from previous studies examining progression from IFG or IGT to diabetes [8, 11, 13, 14]. In contrast with our findings, previous studies from India and South Africa did not detect an association between waist circumference and a progression to diabetes [6, 35]. This disparity in results might be partly explained by the different ethnic groups included in the study populations. In our study, only decreases in BMI and waist circumference during follow-up but not their baseline values determined regression to normoglycemia. This finding is consistent with results of regional cohort studies of individuals with IFG or IGT in Germany [12] or the UK [4].

Interestingly, in multivariate analyses, none of the considered lifestyle factors was significantly associated with glycemic changes. This was also the case for fruit or vegetable intake and consumption of sugar-sweetened beverages or alcohol (data not shown). For physical activity, our null finding is consistent with the majority of previous studies that define glycemic status based on IFG, IGT [4, 6, 7, 9, 10, 15, 17], or HbA1c [17]. Only a few prior studies have investigated the association between dietary behaviors and changes in glycemic status in individuals with prediabetes, and the results have been inconsistent [7, 16]. The same is true for the association between smoking status and changes in glycemic status in individuals with prediabetes [6–10]. Differences in results might be explained by differences in the accuracy of the assessment methods used. In most epidemiological studies, including the present study, lifestyle factors are assessed through self-reporting, which is considered a rather imprecise method and is moreover prone to social desirability and recall bias [36, 37]. Given the high potential for misclassification, existing associations between lifestyle factors and change in glycemic status might not be detected [4]. In the case of physical activity, we also had to combine the assessed 5 categories into a dichotomous variable due to the relatively small sample sizes in certain categories, which might have been too broad to identify existing associations. Of particular relevance to dietary intake, a further explanation for our null finding might be that the influence of individual dietary components is fairly small and therefore difficult to detect. In the present study, values of the diabetes risk score had the expected associations with glycemic changes. These findings could not be fully explained by waist circumference or age (as two main score components) and thus highlight the value of a multifactorial diabetes risk assessment. In addition, a recent interventional study suggests that among individuals with prediabetes, there might be a high-risk phenotype that does not respond to lifestyle interventions such as dietary counseling and exercise [38–40].

Despite their discussed role in diabetes pathophysiology, traffic intensity and mental distress were also not related to glycemic changes among prediabetic adults in multivariate analyses. While we did not find a comparable study on traffic-related exposures, one study showed that mental loss as assessed by the mental component score of the SF-12 questionnaire was associated with a progression to diabetes in adults with IFG or IGT [41].

Similar to our findings, previous studies of individuals with IFG found higher baseline levels [7, 8, 10] or an increase in triglycerides [7] to be associated with a progression in glycemic status. However, the results that show a progression in glycemic status are less consistent for studies that analyze individuals with IFG and IGT together [6, 9, 35]. Only a few previous studies have explored measures of lipid metabolism with respect to a regression in glycemic status from IFG or IGT, and the findings have been inconsistent [9, 10]. The findings of the current study for liver enzymes, i.e., the association of higher GGT and ALT levels with progression in glycemic status, are in line with findings for GGT observed for individuals with IFG or IGT in a South African cohort [35]. With respect to hs-CRP, a study among Chinese adults with IGT found that higher levels of hs-CRP indicated a progression to diabetes [42], whereas no association with glycemic changes was observed in our study.

### 4.2. Limitations

This nationwide cohort study comprised participants from the general adult population in Germany covering a wide age range. However, the follow-up rate was rather moderate and the GNHIES98 participants who took part in the follow-up survey had some different characteristics from those who did not reparticipate or were deceased [24]. To account for differences arising from nonparticipation, a weighting factor was computed following the approach described above [27] and applied to all analyses in the present study.

Despite the application of a weighting factor, our study population might have been healthier compared to other studies. This was reflected by a higher proportion (34%) of individuals who reverted to normoglycemia compared to some other studies. For example, the proportion of participants with a regression was 4.4% based on data from the Whitehall II study, which applied the same definition for prediabetes but had a shorter follow-up time [17]. On the other hand, similar to that in our study, the proportion of individuals with a progression to diabetes was approximately one-fifth of participants in the Atherosclerosis Risk in Communities (ARIC) study, which used the same prediabetes definition and a similar follow-up length [43] as those of our study. One reason for the rather high proportion of individuals who reverted to normoglycemia in our study might be the relatively high proportion of individuals at the lower threshold of HbA1c-defined prediabetes. Thus, in a sensitivity analysis, we applied a stricter definition to identify only *relevant* changes in glycemic status. Nonetheless, the results remained largely unchanged.

Moreover, the proportion of participants with a migration background was rather low, and therefore, ethnicity could not be taken into account as a potential determining factor for glycemic changes [44]. We also had no information on whether participants only temporarily progressed to diabetes or regressed to normoglycemia between baseline and follow-up [45]. Further, we cannot rule out that the observed associations between changes in risk factors and changes in glycemic status in the additional analyses might be biased from reverse causation. Moreover, we did not adjust for multiple testing since analyses were exploratory.

Based on HbA1c measures and information about diabetes diagnosis, the present study permitted the identification of similarities and differences in the progression to diagnosed and undiagnosed diabetes. However, due to the lack of additional markers of glucose metabolism, we were unable to directly compare findings between individuals whose prediabetes was based on HbA1c and those whose prediabetes was defined by glucose criteria (e.g., IFG or IGT). The definition of prediabetes based on either of these measures might be too narrow, and a more refined identification of subgroups at high risk of developing diabetes remains a challenge with high relevance for the development of effective case finding and prevention strategies [38, 39, 46].

## 5. Conclusion

In this nationwide cohort of German adults who were followed for 12 years, approximately one-third of the individuals with HbA1c-based prediabetes reverted to normoglycemia and one-fifth progressed to self-reported diagnosed or HbA1c-based undiagnosed diabetes. Measures of anthropometry and lipid metabolism at baseline or as change during follow-up showed the most consistent associations with changes in glycemic status in both directions. In addition, elevated liver enzymes were related to the future development of undiagnosed or diagnosed diabetes; older age and a family history of diabetes were further associated with future diagnosed diabetes. In contrast with results from previous studies that primarily relied on glucose-based definitions of prediabetic states, the findings of the current study appear to be more variable for metabolic than for anthropometric markers. This difference is not surprising, given that the use of different diagnostic criteria to define glycemic status is likely to represent different pathophysiological states of glucose dysregulation. Our results further support the relevance of the HbA1c criterion in the definition of prediabetes and also demonstrate the need for more refined case finding strategies for diabetes prevention.

## Figures and Tables

**Table 1:** Baseline characteristics of individuals with prediabetes at baseline (*n* = 817) according to HbA1c-defined glycemic status at follow-up.

	Regression to normoglycemia	Remained prediabetic	Progression to	
			Undiagnosed diabetes	Diagnosed diabetes
*n* (%)	300 (33.8%)	364 (46.2%)	52 (7.2%)	101 (12.8%)
Unmodifiable risk factors				
Sociodemographic factors				
Age (years)	48.5 (45.9; 51.1)	49.6 (47.6; 51.7)	**53.8 (50.5; 57.2)**	**57.0 (54.8; 59.2)**
Male sex	53.3 (46.5; 60.1)	61.5 (54.8; 67.8)	48.1 (30.4; 66.2)	**41.6 (31.1; 52.9)**
Educational level				
Low	52.2 (45.0; 59.4)	52.6 (45.6; 59.6)	52.4 (34.2; 70.2)	**71.8 (61.6; 82.0)**
Medium/high	47.8 (40.6; 55.0)	47.4 (40.4; 54.4)	47.3 (29.8; 65.8)	28.2 (18.0; 38.4)
Family history of diabetes				
At least one parent with diabetes	27.1 (19.6; 34.5)	35.4 (29.9; 40.8)	31.1 (10.6; 51.6)	46.7 (34.6; 58.8)
Modifiable risk factors				
Anthropometric markers				
BMI (kg/m^2^)	**26.6 (25.8; 27.3)**	27.5 (27.0; 28.1)	**29.6 (28.5; 30.6)**	**30.4 (29.3; 31.6)**
Waist circumference (cm)	**91.1 (89.1; 93.1)**	94.3 (92.8; 95.7)	**98.6 (94.7; 102.5)**	**101.3 (98.0; 104.5)**
Lifestyle factors				
Any sport activity per week	44.9 (37.5; 52.4)	51.2 (44.3; 58.1)	43.8 (29.1; 59.9)	39.6 (29.8; 49.4)
Smoker at baseline				
Never	36.9 (30.4; 43.4)	38.2 (31.4; 45.0)	51.2 (36.5; 65.9)	43.9 (33.1; 54.7)
Former	16.7 (11.7; 21.7)	24.7 (19.7; 29.7)	26.7 (15.6; 42.1)	25.2 (15.7; 34.8)
Current	46.4 (39.1; 53.8)	37.1 (30.9; 43.3)	**21.8 (11.9; 36.7)**	30.9 (20.7; 41.1)
Intake of whole grains (portions/day)	1.3 (1.2; 1.5)	1.2 (1.1; 1.4)	**1.0 (0.8; 1.2)**	1.1 (1.0–1.3)
Intake of red meat (portions/day)	0.58 (0.55; 0.60)	0.59 (0.57; 0.61)	0.54 (0.50; 0.58)	**0.52 (0.47–0.57)**
Intake of coffee (cups/day)	3.2 (2.9; 3.4)	3.1 (2.8; 3.3)	2.8 (2.4; 3.1)	2.8 (2.4; 3.2)
Residential traffic intensity				
Heavy/extreme residential traffic intensity	25.5 (18.3; 32.7)	23.5 (17.7; 29.4)	28.1 (13.5; 49.6)	**33.5 (22.8; 44.2)**
Mental distress (points)	73.9 (71.6; 76.1)	75.4 (73.3; 77.4)	79.0 (74.2; 83.7)	70.9 (67.0; 74.8)
GDRS (points)	**538 (514; 563)**	575 (557; 592)	**633 (599; 666)**	**690 (661; 720)**
Metabolic markers				
HDL cholesterol (mg/dl)	**56.4 (53.5; 59.4)**	52.4 (50.4; 54.6)	51.3 (47.9; 54.9)	50.3 (47.5; 53.2)
Triglycerides (mg/dl)	**121.9 (111.0; 133.8)**	144.1 (134.2; 154.7)	**187.0 (150.2; 232.9)**	**175.2 (155.0; 198.0)**
GGT (U/l)	**13.9 (12.7; 15.1)**	15.6 (14.6; 16.7)	**18.9 (16.2; 22.1)**	**20.1 (17.2; 23.5)**
ALT (U/l)	12.3 (11.4; 13.2)	12.7 (12.0; 13.5)	**16.0 (13.3; 19.2)**	**15.0 (13.5; 16.5)**
hs-CRP (mg/l)	1.4 (1.1; 1.7)	1.3 (1.2; 1.5)	1.4 (0.9; 2.3)	**2.3 (1.7; 3.1)**

Information is given as weighted proportions (95% CI) for categorical variables, as weighted arithmetic mean (95% CI) for continuous variables, and in case of metabolic markers and predicted 5-year diabetes risk as weighted geometric mean (95% CI). Differences in proportions and means between groups of glycemic status (reference group: remained prediabetic) were assessed by logistic regression and ANOVA. Bold numbers indicate *p* < 0.05.

**Table 2:** Multivariable adjusted odds ratios (95% CI) for associations between risk factors of type 2 diabetes and change in glycemic status during follow-up among individuals with prediabetes at baseline (*n* = 817).

Risk factor at baseline		Regression to normoglycemia	Remained prediabetic	Progression to	
				Undiagnosed diabetes	Diagnosed diabetes
*n* (%)		300 (33.8%)	364 (46.2%)	52 (7.2%)	101 (12.8%)
Unmodifiable risk factors					
Sociodemographic factors					
Age (per year)	Model 1	0.99 (0.97; 1.01)	1.00 (ref)	1.02 (1.00; 1.04)	**1.04 (1.02; 1.06)**
Sex (male vs. female)	Model 1	**0.67 (0.46; 0.98)**	1.00 (ref)	0.66 (0.30; 1.44)	**0.56 (0.33; 0.97)**
Educational level (low vs. middle/high)	Model 1	1.03 (0.69; 1.54)	1.00 (ref)	0.82 (0.38; 1.77)	1.72 (0.90; 3.30)
Family history of diabetes					
At least one parent with diabetes (yes vs. no)	Model 1	0.65 (0.41; 1.03)	1.00 (ref)	0.85 (0.31; 2.35)	**1.82 (1.05; 3.15)**
Modifiable risk factors					
Anthropometric markers					
BMI (per 1 kg/m^2^)	Model 1	0.95 (0.90; 1.01)	1.00 (ref)	**1.09 (1.03; 1.16)**	**1.12 (1.05; 1.19)**
	Model 2	0.95 (0.90; 1.00)	1.00 (ref)	**1.10 (1.02; 1.18)**	**1.13 (1.06; 1.21)**
Waist circumference (per 1 cm)	Model 1	0.98 (0.96; 1.00)	1.00 (ref)	**1.04 (1.01; 1.07)**	**1.06 (1.03; 1.09)**
	Model 2	0.98 (0.96; 1.00)	1.00 (ref)	**1.04 (1.01; 1.07)**	**1.06 (1.03; 1.09)**
Lifestyle factors					
Sport (no sport vs. any sport)	Model 1	1.29 (0.89; 1.88)	1.00 (ref)	1.28 (0.64; 2.55)	1.48 (0.88; 2.50)
	Model 2	1.39 (0.96; 2.01)	1.00 (ref)	1.04 (0.47; 2.27)	0.93 (0.52; 1.65)
Smoker (former vs. never)	Model 1	0.81 (0.49; 1.33)	1.00 (ref)	0.94 (0.37; 2.47)	1.22 (0.62; 2.42)
	Model 2	0.79 (0.48; 1.30)	1.00 (ref)	0.96 (0.39; 2.36)	1.21 (0.59; 2.48)
Smoker (current vs. never)	Model 1	1.40 (0.87; 2.27)	1.00 (ref)	0.57 (0.24; 1.33)	1.42 (0.75; 2.66)
	Model 2	1.29 (0.79; 2.13)	1.00 (ref)	0.59 (0.23; 1.52)	1.58 (0.78; 3.20)
Intake of whole grains (per portion)	Model 1	1.12 (0.90; 1.39)	1.00 (ref)	0.69 (0.46; 1.03)	0.89 (0.63; 1.27)
	Model 2	1.18 (0.95; 1.46)	1.00 (ref)	0.68 (0.44; 1.06)	0.95 (0.64; 1.40)
Intake of red meat (per portion)	Model 1	1.42 (0.41; 4.86)	1.00 (ref)	0.54 (0.09; 3.44)	0.41 (0.05; 3.46)
	Model 2	1.82 (0.52; 6.30)	1.00 (ref)	0.36 (0.06; 2.24)	0.31 (0.03; 3.00)
Intake of coffee (per cup)	Model 1	1.04 (0.92; 1.18)	1.00 (ref)	0.91 (0.77; 1.08)	0.94 (0.77; 1.14)
	Model 2	1.03 (0.90; 1.17)	1.00 (ref)	0.91 (0.74; 1.11)	0.88 (0.71; 1.10)
Residential traffic intensity (heavy/extreme vs. rare/considerable)	Model 1	1.13 (0.71; 1.78)	1.00 (ref)	1.25 (0.49; 3.21)	1.60 (0.99; 2.58)
	Model 2	1.10 (0.70; 1.74)	1.00 (ref)	1.25 (0.51; 3.09)	1.30 (0.76; 2.23)
Mental distress (per 10 points)	Model 1	0.97 (0.85; 1.09)	1.00 (ref)	1.19 (0.93; 1.51)	0.86 (0.72; 1.02)
	Model 2	0.99 (0.87; 1.12)	1.00 (ref)	1.18 (0.92; 1.51)	0.86 (0.72; 1.03)
GDRS (per 10 points)	Unadjusted	**0.98 (0.97; 1.00)**	1.00 (ref)	**1.03 (1.01; 1.06)**	**1.07 (1.04; 1.10)**
Metabolic markers					
HDL cholesterol (per 10 mg/dl)	Model 1	**1.16 (1.01; 1.33)**	1.00 (ref)	0.87 (0.69; 1.08)	**0.74 (0.62; 0.89)**
	Model 2	**1.17 (1.02; 1.35)**	1.00 (ref)	0.90 (0.71; 1.14)	0.85 (0.70; 1.04)
Triglycerides (per 10 mg/dl)	Model 1	0.98 (0.96; 1.01)	1.00 (ref)	**1.04 (1.01; 1.06)**	**1.03 (1.01; 1.05)**
	Model 2	0.99 (0.96; 1.01)	1.00 (ref)	**1.03 (1.01; 1.06)**	1.02 (1.00; 1.04)
GGT (per 1 U/l)	Model 1	1.00 (0.98; 1.02)	1.00 (ref)	**1.02 (1.01; 1.04)**	**1.03 (1.02; 1.05)**
	Model 2	1.00 (0.98; 1.02)	1.00 (ref)	**1.02 (1.00; 1.03)**	**1.03 (1.01; 1.04)**
ALT (per 1 U/l)	Model 1	1.00 (0.97; 1.03)	1.00 (ref)	**1.07 (1.03; 1.12)**	**1.08 (1.04; 1.12)**
	Model 2	1.02 (0.98; 1.05)	1.00 (ref)	**1.06 (1.01; 1.10)**	**1.07 (1.03; 1.10)**
hs-CRP (per 1 mg/l)	Model 1	1.02 (0.99; 1.06)	1.00 (ref)	0.95 (0.84; 1.08)	1.02 (0.98; 1.05)
	Model 2	1.02 (0.99; 1.06)	1.00 (ref)	0.91 (0.76; 1.08)	0.98 (0.95; 1.02)

Bold numbers indicate statistically significant ORs (*p* < 0.05). For all individual risk factors, *model 1* was adjusted for age and sex. For anthropometric markers, *model 2* was additionally adjusted for educational level, lifestyle factors, residential traffic intensity, and mental distress. For lifestyle factors, residential traffic intensity, mental distress, and metabolic markers, *model 2* was additionally adjusted for educational level, anthropometric markers, lifestyle factors, residential traffic intensity, and mental distress.

## Data Availability

The data set used in the study cannot be made publicly available because informed consent from study participants did not cover public access to the data. However, the minimal data set underlying the findings presented in this study is archived in the “Health Monitoring” Research Data Centre at the Robert Koch Institute (RKI) and may be accessed by all interested researchers. On-site access to the data set is possible at the Secure Data Center of the RKI's “Health Monitoring” Research Data Centre. Requests should be submitted to fdz@rki.de.

## Abbreviations

ADA: American Diabetes Association
ALT: Alanine aminotransferase
DEGS1: German Health Interview and Examination Survey for Adults
GGT: Gamma-glutamyl transferase
GNHIES98: German National Health Interview and Examination Survey 1998
GDRS: German Diabetes Risk Score
hs-CRP: High-sensitivity C-reactive protein
IFG: Impaired fasting glucose
IGT: Impaired glucose tolerance.

## Supplementary Materials

Supplementary Materials Supplementary Table 1: multivariable adjusted odds ratios (95% CI) for associations between change in modifiable risk factors of type 2 diabetes and change in glycemic status during follow-up among individuals with prediabetes at baseline (*n* = 817).
