# Supplementary material for: Determinants of Change in Glycemic Status in Individuals with Prediabetes: Results from a Nationwide Cohort Study in Germany
Source: J Diabetes Res. 2018 Oct 14;2018:5703652. doi: 10.1155/2018/5703652 (PMC6204174; doi:10.1155/2018/5703652)
Supplement: Supplementary Materials — Supplementary Table 1: multivariable adjusted odds ratios (95% CI) for associations between change in modifiable risk factors of type 2 diabetes and change in glycemic status during follow-up among individuals with prediabetes at baseline (n = 817). [file 5703652.f1.docx]

Supplementary Table 1. Multivariable adjusted odds ratios (95% CI) for associations between change in modifiable risk factors of type 2 diabetes and change in glycemic status during follow-up among persons with prediabetes at baseline (n=817).

| **Change in risk factor between baseline and follow-up** |  | **Regression to** | **Remained** | **Progression to** | |
| --- | --- | --- | --- | --- | --- |
|  |  | **normoglycemia** | **prediabetic** | **Undiagnosed**  **diabetes** | **diagnosed diabetes** |
| n (%) |  | 300 (33.8%) | 364 (46.2%) | 52 (7.2%) | 101 (12.8%) |
| **modifiable risk factors** |  |  |  |  |  |
| **anthropometric markers** |  |  |  |  |  |
|  | Model 1 | **0.87 (0.78; 0.95)** | 1.00 (ref) | 1.14 (0.98; 1.33) | 1.05 (0.93; 1.19) |
| BMI (per 1 kg/m² increase) | Model 2 | **0.86 (0.79; 0.95)** | 1.00 (ref) | 1.15 (0.98; 1.35) | 1.05 (0.94; 1.17) |
|  | Model 3 | **0.86 (0.77; 0.95)** | 1.00 (ref) | 1.15 (0.99; 1.35) | 1.06 (0.96; 1.18) |
|  | Model 1 | **0.97 (0.94; 1.00)** | 1.00 (ref) | 1.03 (0.99; 1.07) | 1.02 (0.98; 1.05) |
| waist circumference (per 1 cm increase) | Model 2 | **0.97 (0.94; 1.00)** | 1.00 (ref) | 1.03 (0.99; 1.07) | 1.02 (0.99; 1.06) |
|  | Model 3 | **0.96 (0.93; 0.99)** | 1.00 (ref) | **1.04 (1.00; 1.08)** | 1.04 (1.00; 1.08) |
| **lifestyle factors** |  |  |  |  |  |
|  | Model 1 | 1.19 (0.81; 1.74) | 1.00 (ref) | 0.85 (0.44; 1.62) | 0.86 (0.53; 1.39) |
| increase in sport activity | Model 2 | 1.20 (0.82; 1.76) | 1.00 (ref) | 0.78 (0.38; 1.59) | 0.68 (0.40; 1.16) |
|  | Model 3 | 1.03 (0.64; 1.65) | 1.00 (ref) | 0.69 (0.30; 1.59) | 0.62 (0.32; 1.17) |
|  | Model 1 | **0.52 (0.30; 0.90)** | 1.00 (ref) | 1.49 (0.39; 5.75) | 0.47 (0.25; 0.87) |
| starting to smoke | Model 2 | **0.50 (0.28; 0.89)** | 1.00 (ref) | 1.51 (0.47; 4.84) | 0.51 (0.26; 1.02) |
|  | Model 3 | 0.56 (0.30; 1.05) | 1.00 (ref) | 1.18 (0.31; 4.55) | 0.53 (0.23; 1.21) |

| intake of whole grain (per 1 portion increase) | Model 1 | 1.01 (0.90; 1.14) | 1.00 (ref) | 0.96 (0.80; 1.16) | 0.99 (0.83; 1.18) |
| --- | --- | --- | --- | --- | --- |
|  | Model 2 | 1.01 (0.89; 1.14) | 1.00 (ref) | 0.95 (0.78; 1.16) | 0.97 (0.79; 1.18) |
|  | Model 3 | 1.03 (0.91; 1.16) | 1.00 (ref) | 0.89 (0.69; 1.14) | 0.96 (0.78; 1.18) |
| intake of red meat (per 1 portion increase) | Model 1 | 1.47 (0.60; 3.57) | 1.00 (ref) | 1.05 (0.22; 5.08) | 1.50 (0.38; 5.95) |
|  | Model 2 | 1.51 (0.61; 3.77) | 1.00 (ref) | 1.02 (0.21; 4.87) | 1.46 (0.37; 5.69) |
|  | Model 3 | 1.87 (0.67; 5.19) | 1.00 (ref) | 0.75 (0.12; 4.80) | 1.07 (0.25; 4.67) |
| intake of coffee (per 1 cup increase) | Model 1 | 1.00 (0.95; 1.06) | 1.00 (ref) | 0.96 (0.87; 1.05) | 0.97 (0.89; 1.06) |
|  | Model 2 | 1.00 (0.94; 1.06) | 1.00 (ref) | 0.96 (0.87; 1.05) | 0.97 (0.89; 1.06) |
|  | Model 3 | 1.00 (0.94; 1.06) | 1.00 (ref) | 0.95 (0.85; 1.06) | 0.97 (0.88; 1.06) |
| **increase in residential traffic intensity** | **Model 1** | **1.07 (0.63; 1.81)** | **1.00 (ref)** | **0.66 (0.23; 1.93)** | **1.57 (0.70; 3.54)** |
|  | Model 2 | 1.07 (0.64; 1.81) | 1.00 (ref) | 0.59 (0.19; 1.84) | 1.72 (0.80; 3.67) |
|  | Model 3 | 1.15 (0.61; 2.15) | 1.00 (ref) | 0.58 (0.14; 2.46) | 2.15 (0.94; 4.93) |
| **mental distress** (per 10 points increase) | Model 1 | 0.96 (0.84; 1.10) | 1.00 (ref) | 0.93 (0.73; 1.17) | 0.96 (0.77; 1.19) |
|  | Model 2 | 0.95 (0.83; 1.09) | 1.00 (ref) | 0.95 (0.75; 1.20) | 0.98 (0.79; 1.22) |
|  | Model 3 | 0.93 (0.79; 1.10) | 1.00 (ref) | 1.01 (0.77; 1.34) | 0.89 (0.72; 1.10) |
| **GDRS** (per 10 points increase) | unadjusted | 0.98 (0.96; 1.01) | 1.00 (ref) | 1.03 (0.99; 1.07) | 1.02 (0.98; 1.06) |
|  | adjusted for baseline | 0.98 (0.95; 1.01) | 1.00 (ref) | **1.04 (1.01; 1.08)** | **1.06 (1.01; 1.10)** |
| **metabolic markers** |  |  |  |  |  |
| HDL cholesterol (per 10 mg/dl increase) | Model 1 | 1.00 (0.83; 1.20) | 1.00 (ref) | 1.02 (0.70; 1.47) | 1.14 (0.91; 1.44) |
|  | Model 2 | 0.98 (0.82; 1.17) | 1.00 (ref) | 1.03 (0.70; 1.50) | 1.05 (0.83; 1.35) |
|  | Model 3 | 1.17 (0.95; 1.45) | 1.00 (ref) | 0.89 (0.52; 1.51) | 0.88 (0.64; 1.21) |
| triglycerides (per 10 mg/dl increase) | Model 1 | 0.99 (0.98; 1.01) | 1.00 (ref) | 0.97 (0.94; 1.00) | 1.01 (0.97; 1.06) |
|  | Model 2 | 0.99 (0.97; 1.01) | 1.00 (ref) | 0.97 (0.94; 1.00) | 1.02 (0.99; 1.05) |
|  | Model 3 | **0.94 (0.89; 1.00)** | 1.00 (ref) | 1.00 (0.97; 1.04) | **1.05 (1.02; 1.09)** |
| hs-CRP (per 1 mg/l increase) | Model 1 | 0.99 (0.96; 1.02) | 1.00 (ref) | 1.01 (0.99; 1.03) | 1.01 (0.98; 1.04) |
|  | Model 2 | 0.99 (0.95; 1.02) | 1.00 (ref) | 1.01 (0.99; 1.04) | 1.02 (0.99; 1.05) |
|  | Model 3 | 1.00 (0.95; 1.05) | 1.00 (ref) | 1.00 (0.96; 1.04) | 1.02 (0.98; 1.07) |

Bold numbers indicate statistically significant ORs (p<0.05). For all individual risk factors **Model 1** was adjusted for age and sex. For anthropometric markers **Model 2** was additionally adjusted for educational level, lifestyle factors, residential traffic intensity, and mental distress. For lifestyle factors, residential traffic intensity, mental distress and metabolic markers **Model 2** was additionally adjusted for educational level, anthropometric markers, lifestyle factors, residential traffic intensity, and mental distress. **Model 3** was further adjusted for the baseline level of the respective risk factor.

GDRS = German Diabetes Risk Score
